# Exploring the structural landscape of universal stress proteins in *Archaea*

**DOI:** 10.1007/s11274-025-04439-y

**Published:** 2025-06-11

**Authors:** L. Matarredona, M.J. García-Bonete, B. Zafrilla, J. Esclapez

**Affiliations:** Department of Biochemistry and Molecular Biology and Soil Science and Agricultural Chemistry, Faculty of Science, University of Alicante, Alicante, Spain

**Keywords:** Universal stress protein, *Halobacteriota*, *Methanobacteriota*, *Thermoproteota*, Protein structure, Stress

## Abstract

The universal stress protein (USP) superfamily is widely distributed in the three domains of life. These proteins play a key role in the cell response to biotic and abiotic stresses. Although the discovery of the first USP was more than 30 years ago, little is known about its specific function and the molecular mechanisms underlying it. Focusing on the *Archaea* domain, USPs represent one of the mechanisms developed to survive under environmental stresses, such as nutritional deprivation, oxidative stress, or heavy metal toxicity. This review provides a comprehensive understanding of USPs in this domain, with a focus on their structure and function. It delves into the distribution of USPs in several families of *Archaea* and explores the structures of selected USPs, paying special attention to those of halophilic and thermophilic microorganisms. Additionally, the biotechnological implications of USPs in *Archaea* are examined, highlighting their potential in protein engineering and the development of novel biotechnological applications. Understanding the structure and function of USPs will expedite the development of improved industrial processes and contribute to the advancement of innovative medical therapies.

**Supplementary Information:**

The online version contains supplementary material available at 10.1007/s11274-025-04439-y.

## Introduction

Universal stress proteins (USPs), initially discovered in *Escherichia coli* (Nyström and Neidhardt 1992), are widely distributed in organisms from all three domains of life, including plants, fungi, bacteria and archaea, but they are absent in humans (Siegele 2005; Hingley-Wilson et al. 2010). Generally, these proteins help organisms to cope with unfavorable conditions or biotic and abiotic stresses, playing a role in protein scaffolding, biomolecules transport, preventing protein or DNA denaturation, and electron transport (Gustavsson et al. 2002; Kvint et al. 2003).

The best-known USPs are those for *E. coli*. Specifically, a family of six homologous USPs (UspA, UspC, UspD, UspE, UspF, and UspG) has been described in this microorganism. Based on sequence homology and structural characteristics, *E. coli* USPs are classified into four different classes: Class I (UspA, UspC, UspD) lacks an ATP binding motif, whereas Class II, which includes UspF and UspG, contains ATP binding motifs. UspE possesses two tandem USP domains E1 and E2, which are categorized as Class III and IV, respectively (Kvint et al. 2003; Vollmer and Bark 2018). It has been demonstrated that each USP class contributes to different stress tolerance: Class I USPs are involved in processes of oxidative stress resistance, nutrient starvation (carbon, nitrogen, phosphate and sulphur), heavy metals tolerance and iron scavenging; Class II USPs participate in cellular adhesion, cell motility and swimming processes and also in oxidative stress protection; Class III involves signalling molecules that detect stress and activate adaptive responses; Class IV ensures cellular survival and adaptation through synergistic interactions among different USPs under stress (Nabi et al. 2024).

USPs in other bacteria exhibit structural and functional diversity, often through fusion with catalytic motifs such as protein kinases, ion exchangers, and permeases (Luo et al. 2023). These adaptations not only enhance stress tolerance but also increase pathogenicity. For example, in *Acinetobacter baumannii*, one of the greatest threats to global public health, UspA protects against oxidative stress, low pH and respiratory toxin (Elhosseiny et al. 2014) and in *Salmonella typhimurium* UspA makes an important contribution to its in vivo virulence (Liu et al. 2007). Similarly, *Listeria monocytogenes* USPs confer resistance to oxidative and acid conditions (Seifart Gomes et al. 2011), and *Mycobacterium tuberculosis* Rv2623 is involved in bacterial persistence and chronic infection (Drumm et al. 2009; Glass et al. 2017).

Like the bacterial USPs, plant USPs have multiple functions related to environmental stress protection. The model plant *Arabidopsis thaliana* possesses more than 40 USPs, which act as molecular chaperone, under heat and oxidative stress, RNA chaperone under cold stress or regulator of ROS production under anoxia (Chi et al. 2019). In *Solanum lycopersicum* the USP known as SIRd2 is a phosphorylation target of the calcineurin B-like interacting protein kinases (CIPKs). It confers resistance to osmotic and salt stress (Gutiérrez-Beltrán et al. 2017). In addition, it has also been described that plant USPs regulate the photosynthetic efficiency under drought conditions through redox regulation (Loukehaich et al. 2012).

Despite significant research in bacteria and plants, the physiological roles of USPs in *Archaea* remain largely unexplored, even though two structures have been described in hyperthermophilic microorganisms. The structure of *Methanococcus jannaschii* MJ0577 was the first USP structure solved in *Archaea* (Zarembinski et al. 1998) Its ATP binding site was coordinated by a divalent cation and composed of the following residues: G_127_-SH-G_130_-(9X)- G_140_. MJ0577 alone showed negligible ATPase activity, but ATP hydrolysis was detected in the presence of *M. jannaschii* cell extract suggesting a regulatory role as an ATP-dependent molecular switch. *Archaeoglobus fulgidus* AF0826 USP was described as a dimeric protein whose structure contains a nearly canonical ATP binding motif that accommodates of the adenosine part of ATP (Tkaczuk et al. 2013). Although these studies described USP structures in *Archaea*, their function remains still unknown. In recent years, studies with *Sulfolobus acidocaldarius* USPs have elucidated some of the roles of USPs (Ye et al. 2020a, b). This aerobic thermoacidophile archaeon has three different USP encoded in its genome. SaUspA forms a stable homodimer, conserves the ATP binding motif, and interacts with the serine/threonine phosphatase PP2A, a eukaryotic PP2A catalytic subunit homolog. This interaction was enhanced by ATP binding and stimulates PP2A activity, suggesting a regulatory role in cellular signalling. Deletion mutants of SaUspsA revealed that this USP was involved in nitrogen starvation and salinity stress response acting as a stress modulator via PP2A interaction (Ye et al. 2020a). Focusing on halophilic *Archaea*, bioinformatic analysis of USPs in six halophilic archaeal families (*Halococcaceae*,* Halorubraceae*,* Natrialbaceae*,* Haloferacaceae*,* Haloarculaceae*,* and Halobacteriaceae*) revealed that most halophilic USPs contain a single USP domain (140–160 amino acids). However, the presence of the USP domain fused with other catalytic motifs, such as amino acid permease or a Na^+^/H ^+^ exchanger, is also frequent. These domain combinations suggest functional diversification, aiding in stress resistance. Strikingly, several genera, including *Halorubrum*,* Haloferax*, and *Haloarcula*, have a relatively high number of USPs (1939, 1301, and 970, respectively), reinforcing their essential response to osmotic stress and cellular homeostasis (Matarredona et al. 2020). In addition, recent studies on *Haloferax mediterranei* have highlighted the complexity of USP expression under stress, suggesting a coordinated response involving multiple USPs to modulate growth and improve survival under adverse conditions (Matarredona et al. 2024). Transcriptional expression of the 37 annotated USPs in the *Hfx. mediterranei* genome was examined under three different stress conditions. A clear relationship between the number of USPs and the growth phase was observed, but it was difficult to associate the expression of a single USP with a particular type of stress. Besides, approximately one-third of all USPs remained expressed regardless of the stressor. These results suggested that the USP system acts as a recruitment mechanism, where the number of stress proteins expressed increases depending on the strength of the stressful stimulus. Three *Hfx. mediterranei* USPs were studied in detail (USP5, USP21, and USP28), showing that all three form stable homodimers. The physiological characterization of the overexpression strains (USP5-HM26, USP21-HM26, and USP28-HM26) under various stress conditions revealed significant changes in growth kinetic parameters, particularly in the presence of nitrate as nitrogen source, as well as in low and high salinities, and under oxidative stress (Matarredona et al. 2024).

Although further research is needed to enhance our understanding of USP functions and their molecular mechanisms, available data highlight the biotechnological potential of these proteins across various fields. Understanding the structure, distribution, and function of USPs in *Archaea* could provide valuable insights into their adaptation strategies and open up new avenues for industrial and medical applications. This review will focus on the occurrence of USPs in *Archaea*, examining their structural features, potential functions, and the promise they hold for future biotechnological innovations.

### Distribution of USPs in *Archaea*

Universal Stress Proteins (USPs) in *Archaea* play a significant role in adapting to extreme environmental conditions, offering insights into these organisms’ survival mechanisms. This section examined the distribution of USPs across the key archaeal *phyla*: *Halobacteriota*,* Methanobacteriota*, and *Thermoproteota*, as classified in the List of Prokaryotic names with Standing in Nomenclature (LPSN) database. These *phyla* encompass families that thrive in various extreme environments—such as hypersaline (halophiles), anaerobic (methanogens), and high-temperature (thermophiles) habitats. For this study, an analysis of the USPs found in selected archaeal families was performed using the UniProt database. The number of annotated USPs per family was obtained, and these values were normalised by the number of species classified in each family to create a USP per-species ratio. This approach allows comparisons to be made about the number of USPs in different families that are independent of species richness.

The differences in the number of USPs across these *phyla* provide valuable insights into the stress-response mechanisms that have evolved in *Archaea* to deal with various environmental pressures. USPs are known to play a crucial role in protecting cells from damage caused by oxidative stress, heavy metal toxicity, nutrient starvation, osmotic stress, and heat shock (Vollmer and Bark 2018; Ye et al. 2020a; Matarredona et al. 2024). However, in contrast to the extensive research on USPs in bacteria and plants (Nabi et al. 2024), studies on archaeal USPs are limited to a few well-studied species, specifically *M. jannaschii*, *S. acidocaldarius*, *A. fulgidus*, and *H. mediterranei*. The distribution of USPs could reflect the diversity of environmental challenges these organisms face, highlighting their ability to adapt and thrive in such hostile conditions. To make fair comparisons across families with different numbers of species, USP per-species ratio were calculated (Table 1). This USP per species ratio enables us to understand better how much each family invests in stress-response mechanisms, independent of species richness.

**Table 1:** Number of annotated USPs across *Halobacteriora*, *Methanobacteriota* and *Thermoproteota* archaeal *phyla*

Archaeal phyllum	Family	USP/Number species
***Halobacteriota***		
	*Archaeoglobaceae*	104/102
	*Haladaptataceae*	232/38
	*Haloarculaceae*	2202/224
	*Halobacteriaceae*	428/65
	*Halococcaceae*	224/20
	*Haloferacaceae*	4884/336
	*Natrialbaceae* *Natronoarchaeaceae* *Halorutilaceae* *Methanocellaceae* *Methanocorpusculaceae* *Methanomicrobiaceae* *Methanonatronarchaeaceae* *Methermicoccaceae* *Methanotrichaceae*	3673/166 187/4 20/5 48/8 10/152 216/35 18/7 2/9 165/150
***Methanobacteriota***		
	*Methanobacteriace A* *Methanothermace B* *Methanopyraceae*	417/751 2/1 6/9
***Methanobacteriota*** **a**		
	*Methanocaldococcaceae*	17/18
	*Methanococcaceae*	19/53
***Methanobacteriota*** **B**		
	*Methanofastidiosaceae* *Thermococcaceae*	28/19 209/161
***Thermoproteota***		
	*Methanomethylicaceae* *Conexivisphaeraceae* *Nitrosocaldaceae* *Nitrosopumilaceae* *Nitrososphaeraceae* *Caldarchaeaceae* *Calditenuaceae* *Thermofilaceae* *Thermoproteaceae* *Acidilobaceae* *Desulfurococcaceae* *Fervidicoccaceae* *Pyrodictiaceae* *Sulfolobaceae*	34/35 2/9 3/17 569/617 184/167 12/13 1/17 5/32 87/40 24/97 52/67 15/13 27/26 231/190

In the *Halobacteriota phylum*, significative variability was observed in USP counts across families, suggesting differential adaptation strategies. *Haloferacaceae* exhibits the highest USP count (4,884 USPs across 336 species), suggesting a complex and adaptable stress-response system, likely due to fluctuating salinity in its habitat. This family includes *H. mediterranei*, whose 37 USPs have been studied under different environmental conditions, revealing diverse responses to osmotic stress and nutrient deprivation (Matarredona et al. 2024). Other families, such as *Haloarculaceae* and *Natrialbaceae*, also display high USP counts (2,202 and 3,673, respectively), which reflects the high diversity within these families and their adaptation to variable salinity.

In contrast, *Archaeoglobaceae*, with 104 USPs across 102 species, demonstrates one of the lowest USP per-species ratios (1.02), possibly reflecting adaptation to more stable conditions where extensive stress-response proteins are unnecessary, or suggesting that alternative mechanisms may exist to cope with detrimental conditions. The *Natronoarchaeaceae* family, with 187 USPs distributed across four species, shows the highest USP per-species ratio (46.75), suggesting a significant reliance on USPs. This reliance might be due to the extreme variability in environmental conditions. Given that members of this family are likely exposed to fluctuating conditions, such as high salinity and alkalinity, the high number of USPs per species may support a broad range of cellular responses, enhancing their resilience to environmental stressors. The elevated USP count per species in *Natronoarchaeaceae* may support a broad range of cellular responses, enhancing resilience to environmental stressors and suggesting a sophisticated stress management system that remains unknown.

The *Methanobacteriota phylum*, which includes methanogenic archaea adapted to anaerobic environments, displays a wide range of USP counts and ratios across its families. This variability likely reflects the diverse ecological niches and adaptive strategies employed by methanogens to thrive in environments with limited oxygen and variable nutrient availability (Wen et al. 2017). For instance, *Methanobacteriaceae*, with 417 USPs across 751 species, has a low USP per-species ratio (0.55), suggesting that these microorganisms may inhabit relatively stable anaerobic environments where broad stress-response mechanisms are less critical, or they have different mechanisms to cope with stress conditions. *Methanocaldococcaceae* and *Methanococcaceae* exhibit very low USP counts relative to species diversity, indicating limited reliance on these proteins. *Methanocaldococcaceae* has 17 USPs for 18 species (ratio of 0.94), while *Methanococcaceae*, with only 19 USPs across 53 species, has one of the lowest ratios (0.36) in this group. Such low values could imply minimal reliance on USPs, as these organisms may have developed specialized adaptations to consistent anaerobic conditions. In *Methanobacteriota* B, *Methanofastidiosaceae* has 28 USPs across 19 (ratio of 1.47), while *Thermococcaceae* has 209 USPs across 161 species (ratio of 1.3).

The *Thermoproteota phylum*, primarily composed of thermophilic archaea, exhibits considerable variability in USP counts and ratios, reflecting distinct adaptive strategies that extend beyond thermal tolerance. This diversity reflects the different ecological niches and adaptive strategies of these organisms, especially in managing stressors beyond temperature, such as pH fluctuations and nutrient limitations. Higher USP counts are observed in families such as *Thermoproteaceae* (87 USPs across 40 species, with a ratio of 2.17), suggesting a better adaptation to changing environmental factors. Similarly, *Sulfolobaceae*, which includes well-studied extremophiles like *S. acidocaldarius*, has 231 USPs across 190 species, yielding a ratio of 1.21. In contrast, other families within *Thermoproteota*, such as *Nitrosocaldaceae* (3 USPs across 17 species, ratio of 0.18) and *Calditenuaceae* (1 USP across 17 species, ratio of 0.06), possess minimal USP counts. This minimal investment implies that these organisms inhabit highly stable thermal environments, where a broad array of USPs may be unnecessary. Their reduced reliance on USPs could indicate a specialization for consistent, high-temperature conditions, with other proteins, such as heat shock proteins (HSPs), fulfilling critical roles in maintaining cellular stability.

**Fig. 1:**
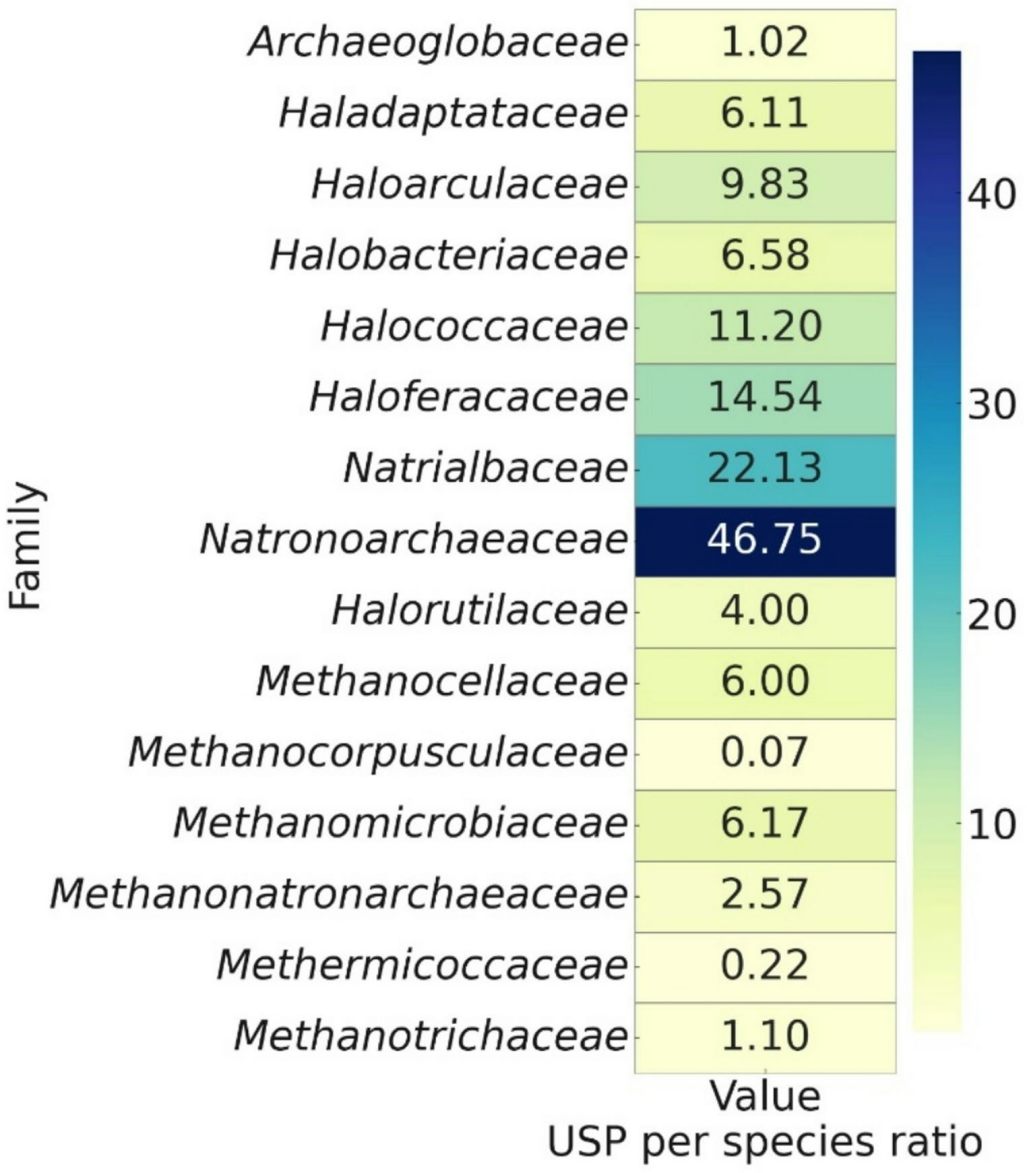
Heatmap of the ratio of USPs per-species in *Archaea* families

The heatmap emphasizes the density of USPs per species across archaeal families (Fig. 1), illustrating distinct adaptive strategies. High ratios in families such as *Methanocorpusculaceae* and *Haloferacaceae* indicate a strong reliance on USPs, potentially due to the fluctuating and extreme environmental conditions these organisms face. Conversely, low ratios in families such as *Methanocaldococcaceae* and *Methanobacteriaceae* suggest a reliance on specialized proteins or stable habitat conditions, thereby reducing the need for extensive USP-based stress responses.

### Archaeal USP domain structure analysis

Archaeal USPs exhibit considerable structural diversity, with various domain combinations that may represent specialized adaptations to extreme environments, such as high salinity, elevated temperatures, and anaerobic conditions. Analysis of USPs across three major archaeal *phyla*—*Halobacteriota*, *Methanobacteriota*, and *Thermoproteota*—reveals distinct patterns of domain structures and distribution, suggesting that these proteins play crucial roles in enhancing stress resilience in specific ecological niches.

The structural domains of USPs typically consist of conserved regions made up of 140–160 amino acids and are present either as single-domain proteins or fused with additional structural domains. Common architectures in archaeal USPs include single USP domains, tandem repeats, and combinations with other functional motifs, such as amino acid permeases and cation/H^+^ exchangers. These configurations suggest functional differentiation, potentially enhancing archaeal resilience by integrating stress-sensing and regulatory roles with membrane transport or ATP-binding activities. This structural variety within archaeal USPs may thus play a critical role in their capacity to endure and respond to environmental stresses.

The *Halobacteriota* phylum exhibits the highest structural diversity, with families like *Haloferacaceae*, *Natrialbaceae*, and *Haloarculaceae* dominating the landscape (Table 2). These families, particularly *Haloferacaceae*, display a wide diversity of USP domain structures, including singular USP domain, tandem USP-USP structures, and more complex configurations involving other catalytic domains such as the amino acid permease (AA_permease) and Na^+^/H^+^ exchanger.

This structural diversity likely equips *Halobacteriota* with the flexibility required to adapt to the highly variable osmotic pressures and ionic concentrations characteristic of hypersaline environments. Moreover, the elevated USP counts observed in families like *Natronoarchaeaceae* and *Natrialbaceae* suggest a significant evolutionary investment in these proteins, possibly as an adaptive strategy to withstand extreme salinity fluctuations and periodic desiccation.

The *Methanobacteriota phylum*, which comprises methanogenic archaea adapted to anaerobic environments, exhibits relatively low diversity and complexity in USP domain configurations (Table 2). Simple structural types, including single USP domains and basic tandem configurations, primarily characterize families such as *Methanobacteriaceae* and *Methanomicrobiaceae*. This structural simplicity suggests that *Methanobacteriota* may have reduced reliance on broad-spectrum stress-response proteins, likely due to the more stable and consistent conditions found in anoxic environments where they predominantly reside.

Notably, some *Methanobacteriota* families, such as *Methanomicrobiaceae*, contain unique domain combinations like the light-independent protochlorophyllide reductase subunit B-like C-terminal domain (LI-POR_suB-like_C) linked with a USP domain. These automatic annotation results are unusual, as the LI-POR_suB-like_C domain has been described in enzymes from cyanobacteria, such as *Synechocystis* or red algae (Dong et al. 2020). In other non-photosynthetic organisms, this domain is associated with oxidation-reduction reactions (Chernomor et al. 2021). Consequently, this specialized configuration in *Methanomicrobiaceae* may support metabolic pathways involved in methanogenesis, highlighting a functional adaptation to their anaerobic lifestyle. Additionally, *Methanobacteriaceae* includes a domain arrangement with a sodium/calcium exchanger, potentially providing ion homeostasis in environments with variable mineral compositions. These unique domain architectures could demonstrate how evolutionary pressures have refined USP structures in the phylum *Methanobacteriota*, enabling these organisms to adapt to environmental challenges by developing specific structural adaptations rather than possessing a wide variety of USP types.

The *Thermoproteota phylum*, which comprises thermophilic archaea adapted to extremely high temperatures, exhibits a unique pattern in its USP structures. Remarkably, all families within this group contain USPs characterized either by a single USP domain or by simple tandem arrangements of two USP domains (USP-USP) (Table 2). Unlike the other two *phyla*, no catalytic domains have been identified in the USP of microorganisms belonging to the *Thermoproteota phylum*. This limited structural diversity could suggest that these organisms have evolved in a specialized way to enable them to endure extreme thermal environments.

This is the first work that analyses the diversity of USP domain structures across *Archaea*. These insights could help guide future research into how USPs support archaeal survival and resilience, with potential applications in biotechnology.

**Table 2:**
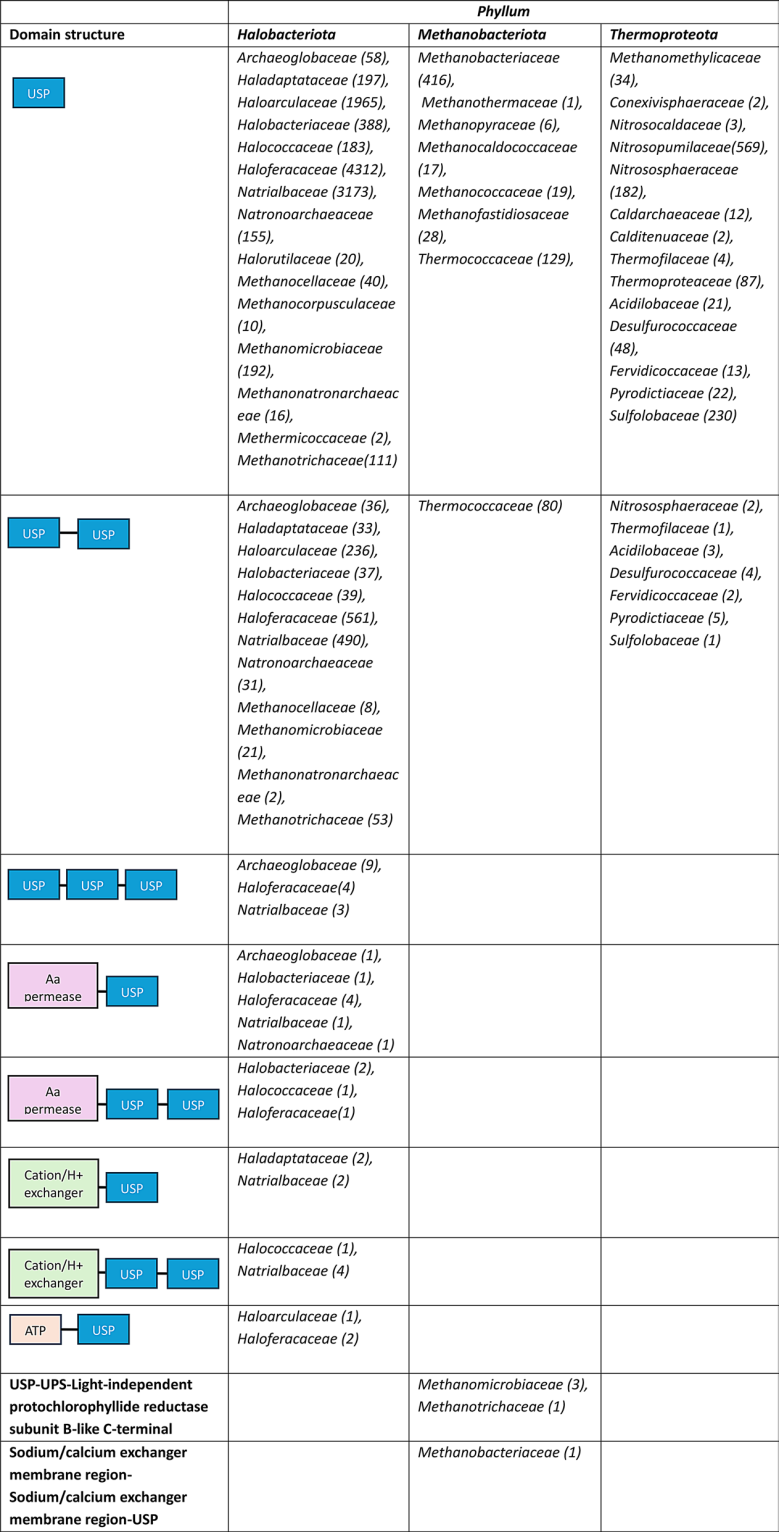
Distribution of domain structures in USPs across *Halobacteriota*,* Methanobacteriota*, and *Thermoproteota* families

### USPs oligomerization state

Generally, USPs in bacteria, archaea and plants exhibit a dimeric quaternary structure, which appears to support their stability and functionality under stress conditions. In *Archaea*, the homodimeric structure has been observed in *M. jannaschii* MJ0577 and *A. fulgidus* AF0826, where studies using techniques like size-exclusion chromatography and dynamic light scattering have consistently demonstrated this dimeric arrangement (Zarembinski et al. 1998; Tkaczuk et al. 2013). In addition, both enzymes contain the ATP binding motif although ATP hydrolysis has not been confirmed in vitro suggesting the requirement for one or more soluble components to stimulate ATPase activity. USP AF1760 from *A. fulgidus* is a USP-tandem protein which form another dimer of tandem “dimers”, the resulting protein contains four USP domains in total. This oligomerization state has also described in other tandem fusion USPs, such as *Thermus thermophilus* HB8 TTHA0350 (Iino et al. 2011). The X-ray structure of the TTHA0350 protein showed that the N-terminal and C-terminal domains bind one ATP, although the ATP-binding motif could not be detected on sequence alignment (Iino et al. 2011).

Dimeric structures have also been described in USP5, USP21, and USP28 from *H. mediterranei*, which form homodimers with molecular weights of 46, 32, and 36 kDa, respectively (Matarredona et al. 2024). Sequence analysis reveals that USP5 and USP21 contain a putative ATP binding motif but not USP28. The USP TeaD from the halophilic bacterium *Halomonas elongata* showed different oligomerization state depending on the concentration of ATP. In the absence of ATP, TeaD is a homodimer of 32 kDa. Still, incubating the protein with ATP resulted in a higher oligomeric state, corresponding to a tetramer with a molecular weight of 62 kDa. This ATP-dependent oligomerization might have a functional role in the regulatory mechanism (Schweikhard et al. 2010).

Apart from dimeric and tetrameric structures, USPs also exist in other native structures, including monomers, trimers and oligomeric complexes formed by combinations of USP proteins (Jung et al. 2015). The oligomeric state switches in response to various parameters, such as external stresses or intracellular ATP concentrations, and appears to underlie some regulatory mechanisms.

Overall, the oligomerization state of USPs across the three domains of life is likely a key factor in modulating their biochemical and physiological functions, including ATP-binding affinity, protein-protein interactions, and activity regulation, which may determine their role in adapting to cellular stress.

### USP structure prediction in *Archaea*

**Fig. 2:**
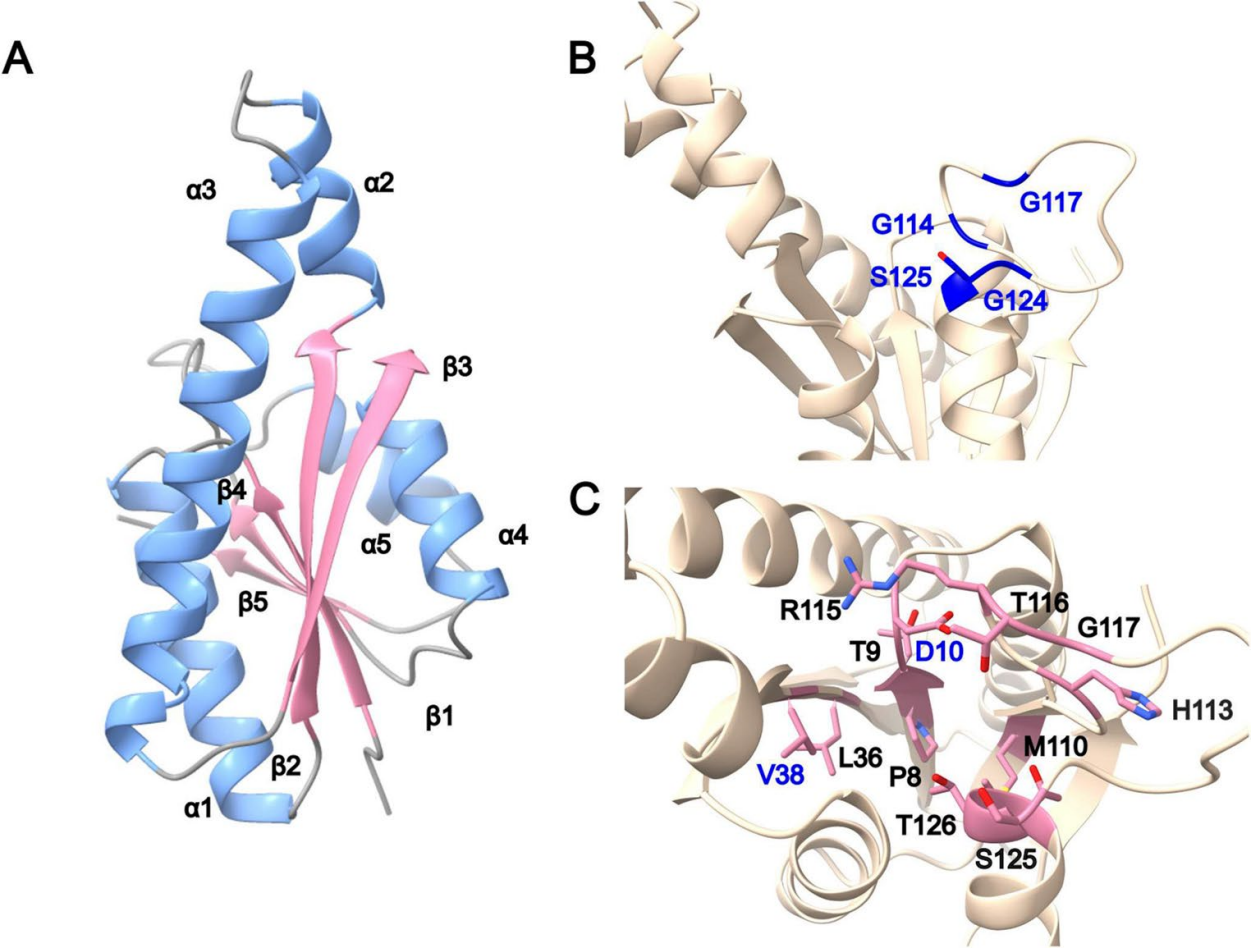
I3R355 single USP protein structure model generated with Alphafold. **(A)** Secondary structure representation. In pink the 5 β-sheet core of the USP domain and in blue the 5 α-helix, including the α_2_ which it is not always present in all the USP. **(B)** ATP binding domain representation; A-2X-G-**9**X-G-(S/T), for I3R355. **(C)** ATP binding pocket including the amino acids involved in the interaction through their side chains or peptide bond

**Fig. 3:**
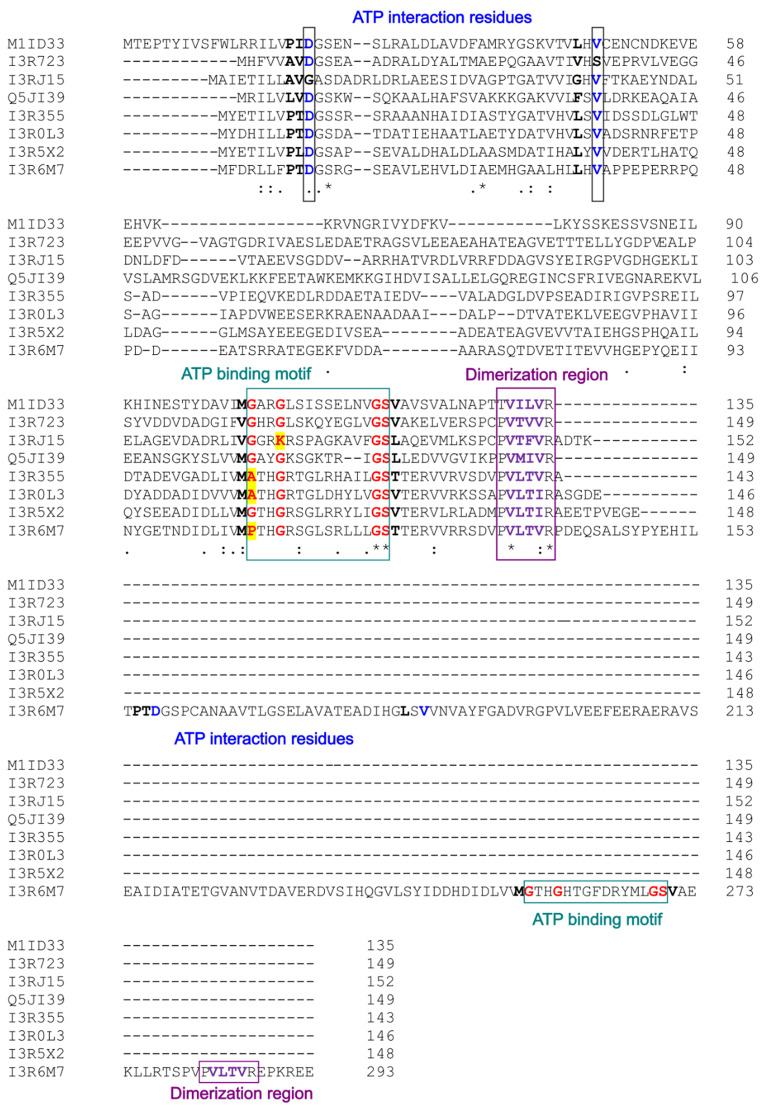
Sequence alignment of single USP and USP-USP (I3R6M7) proteins from *Archaea* using ClustalW omega. In red are represented the residues involved in the ATP binding motif (green square). In black are represented residues involved in the interaction with the ATP according to the PDB-ID 1mjh (Madeira et al. 2024) and in blue are the most critical residues. In purple are represented the residues involved in the dimerization of USP domains and the formation of the antiparallel β-sheet

Single-USP proteins consist of a globular structure with five parallel β-sheets (β1- β2- β3- β4- β5) and 4 or 5 α-helix (α1-“α2”-α3-α4-α5) depending on the specie (Fig. 2A). This extra helix (α2, Fig. 2A) is observed in particular species of *Halobacteriota* and *Methanobacteriota* in addition to a longer α3 helix than in *Thermoproteota* species. This difference is due to a shorter sequence between the β2 sheet and the α3 helix in *Thermoproteota* (Figure S1). The USP domain proteins also presents a representative motif, G-2X-G-9X-G-(S/T) (Tkaczuk et al. 2013), that allows proper arrangement of the tertiary structure to allocate an ATP binding pocket and ion binding site (Fig. 2B). In some USP proteins, this motif can degenerate presenting a larger or shorter number of non-specific residues between the specific glycine residues (G-2X-G-5/12X-G-(S/T) (Tkaczuk et al. 2013), or in some case a substitution of the first glycine (which is at the end of the β4-strand) by alanine or proline (I3R355, I3R6M7 Fig. 3). Alphafold models (e.g., I3R355, I3R6M7) (Mirdita et al. 2022; Varadi et al. 2024) suggest that several of these USP proteins with degenerative motifs still maintain the tertiary structure of the ATP binding pocket, suggesting the possibility of binding ATP and an ion (Fig. 2B). Other USP proteins (e.g., I3R1J5, IR3R723) (Mirdita et al. 2022; Varadi et al. 2024) do not contain this complete motif, suggesting its inability to bind with ATP. Although this motif is essential in forming the ATP binding pocket, other residues outside this motif are involved in binding the ATP and ions. Considering the crystallographic structure of a single USP protein from *M. jannaschii* (PDB-ID 1mjh) (Madeira et al. 2024) where ATP is bound, several residues have been identified in the interaction with the ATP molecule. Residue D12 and V30 (reference molecule I3R355) are essential in this interaction and probably in its hydrolyzation (Fig. 2C). Sequence alignment performed with ClustalW Omega shows these differences in ATP binding motif and essential residues (Fig. 3).

**Fig. 4:**
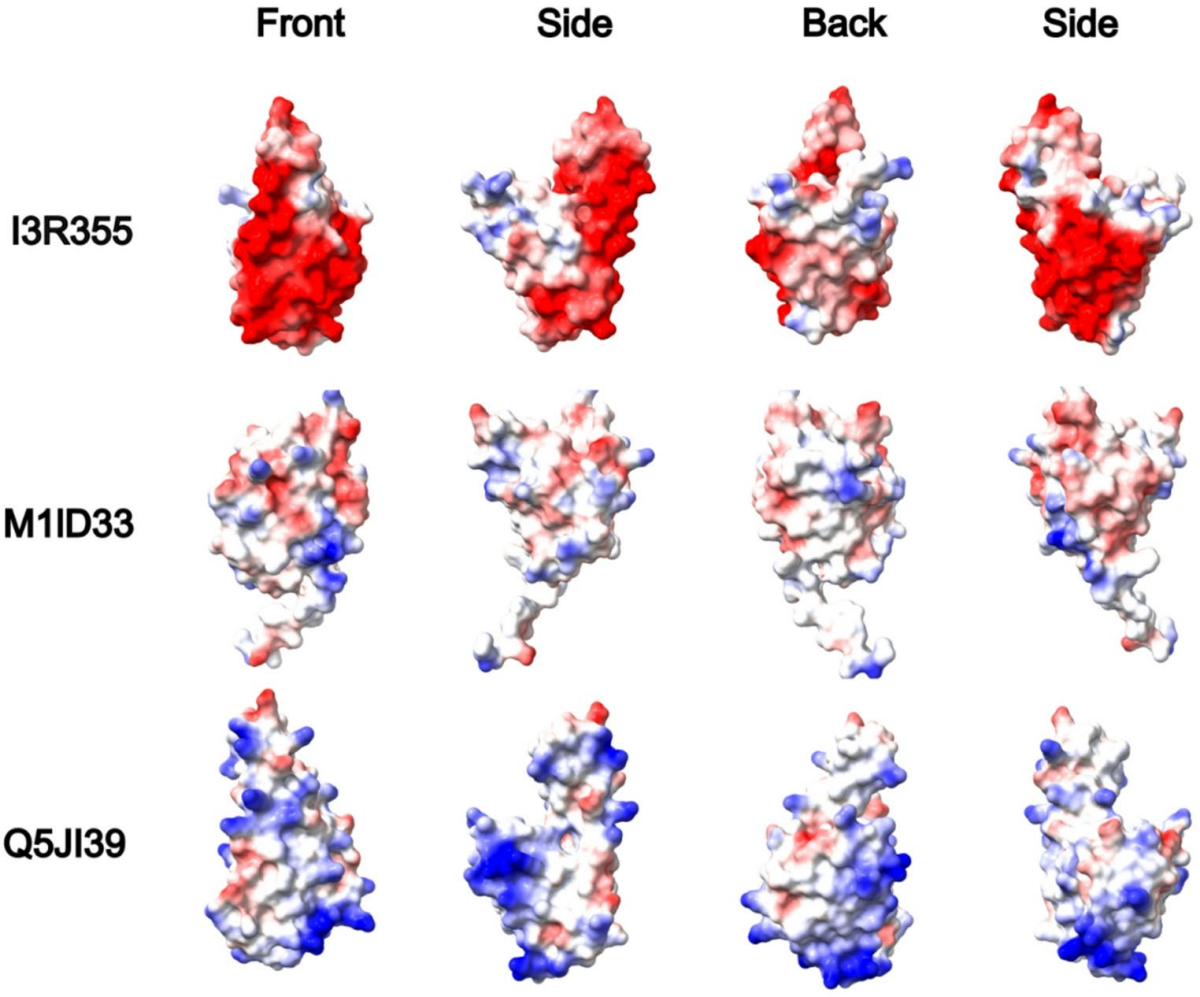
Surface electrostatic potential representation of single USP proteins from each group. Halobacterium proteins present much higher negatively charged surface in comparison with other non-halophilic species. The M1ID33 protein model presents a small tail at the bottom of the structure that corresponds to the N-terminus, which is flexible and does not contribute to the core structure. Red: negative charge residues, blue: positive charge residues and white: non-charge residues

Depending on the microorganism host, the USP proteins can differ in size and surface charge. In Figure S1, we compared three single USP protein from *Halobacteriota*,* Methanobacteriota* and *Thermoproteota* (I3R355, MD1ID33 and Q5JI39, respectively), suggesting a more compact domain for MD1ID33 than for I3R355 and Q5JI39. In Fig. 4, we compare the USP domain surface and their electrostatic potential, showing that halobacterium species present a much higher negatively charged surface (red) than other microorganism groups. The origins of the decrease in nonpolar surface in halophilic proteins, and one of the most striking differences between the surfaces of the halophilic and nonhalophilic enzymes, is a significant reduction in the percentage of hydrophobic surface accessible area due to lysine side chains as has been described in glucose dehydrogenase from *H. mediterranei* (Britton et al., 2006).

**Fig. 5:**
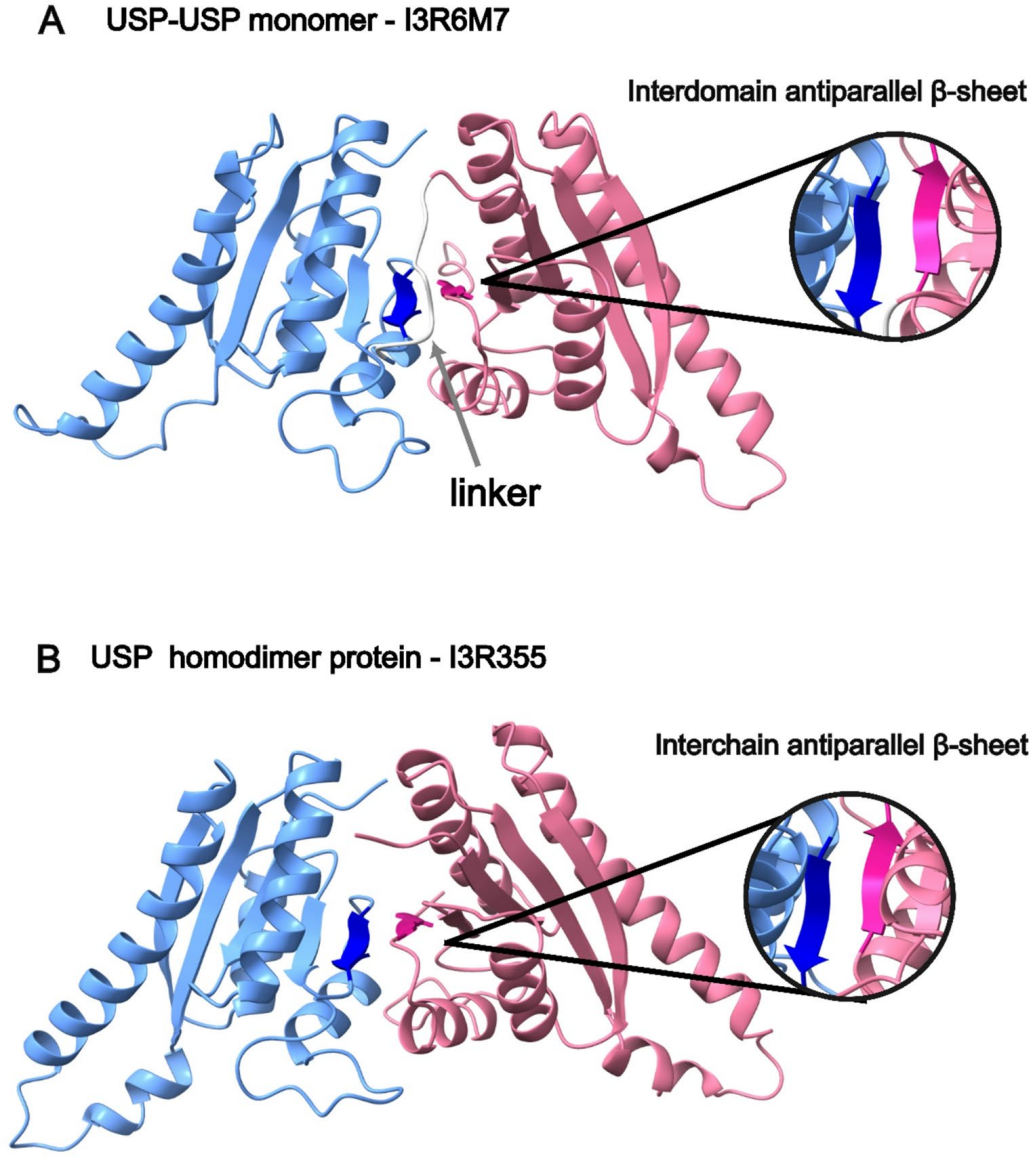
USP domain interactions. **(A)** USP-USP protein structure of I3R6M7 predicted by Alphafold. One domain is represented in blue, the other in pink and the linker between domains is represented in grey. The antiparallel β-sheet involved in the domain interaction is represented in darker colours. **(B)** Single protein structure of I3R355 predicted as a dimer by Alphafold. Both structures present the antiparallel β-sheet between the two USP domains, regardless of whether they are single or double USP domain proteins. One subunit is represented in blue, and the other in pink. The antiparallel β-sheet involved in the oligomerization is represented in darker colours

Several microorganisms also present USP-USP proteins, which contain two USP domains connected through a linker (Fig. 4). According to previous crystallography structures (PDB-ID: 5vb0) (Xu et al. 2016) and Alphafold models predictions, USP domains interact by forming an antiparallel intradomain β-sheet (one from each domain; Figs. 3 and 5A). Both domains can also contain the ATP binding domain (as in the example described in Figs. 3 and 5A) suggesting the possibility of binding two ATP molecules simultaneously. In addition, upon closer examination of their structure and symmetry in the available experimental structures at the Protein DataBase, these proteins may be able to oligomerize into dimers, allowing the simultaneous binding of four ATP molecules and the possibility of allosteric regulation.

Single USP protein models have also suggested the possibility of dimerization and tetramerization. A dimeric model generated with Alphafold2 from the single USP-domain protein, I3R355, shows the formation of an antiparallel interchain β-sheet for dimer formation similarly to that of the USP-USP proteins (Fig. 5B). A tetrameric model also suggests the possibility of tetramerization of single USP proteins and the formation of heterooligomers (Figure S2).

The models studied in this manuscript are generated by artificial intelligence. Although they show a high level of confidence, domain oligomerization and ATP binding in degenerative motifs still need to be confirmed experimentally.

### Biotechnological applications

USPs play an essential role in the survival and adaptation of organisms to diverse environmental stresses. Their wide distribution and functional versatility in pathogens, plants, bacteria and archaea position them as valuable tools in biotechnology.

In pathogens, such as *M. tuberculosis* (Banerjee et al. 2024) or *S. typhimurium* (Liu et al. 2007), USPs enable them to adapt to the conditions found in the human niche, including oxidative or osmotic stress, nutrient starvation or acid stress, thereby facilitating infection. Similarly, bacterial USPs respond to various stresses, including nutrient deprivation, osmotic stress, and exposure to heavy metals (Kvint et al. 2003). On the other hand, USPs in plants are involved in a wide range of stress responses, including drought, salinity, heat, and pathogen attacks (Chi et al. 2019). In the case of *Archaea*, USPs are directly related to their adaptation to extreme environments such as high salinity. Recently, the interaction of SaUspA with threonine/serine phosphatase PP2 has been described (Ye et al. 2020a, b).

The versatility of USPs across different organisms offers numerous biotechnological applications. Focusing on *Archaea*, further investigation into their structural characteristics, putative functions, protein-protein interactions, and molecular mechanisms could unlock new applications in biomedicine, agriculture and bioremediation. In biomedicine, novel archaeal USPs structures could be used to develop antimicrobial therapies as *Archaea* and *Eukarya* organisms share replication, transcription processes, and molecular machinery. In general, halophilic archaea exhibit an increase in USP encoded in their genomes, which appears to be related to the osmotic stress. It has been suggested that the basal expression of a minimal number of USPs could be attributed to the constant need for a high salt concentration, which is crucial for the survival of halophilic organisms like *H. mediterranei* (Matarredona et al. 2024). Following this hypothesis, introducing or increasing USP genes in plants could enhance their salinity tolerance during droughts, which are increasing because of climate change. Archaeal USPs could be useful in industrial processes or environmental bioremediation, where archaea can be engineered to enhance their tolerance under harsh conditions or to absorb heavy metals more effectively in contaminated environments, respectively. However, although the number of USPs identified in *Archaea* has increased significantly, their mechanisms of action remain largely unknown, highlighting the need for further research to explore their potential applications fully.

## Conclusion

The distribution and density of USPs in *Archaea* reflect adaptive strategies to extreme environments. Halophilic families, such as *Haloferacaceae*, have high levels of USPs, supporting survival in fluctuating salinities, whereas methanogens, such as *Methanobacteriaceae*, have fewer USPs, relying on stable anaerobic conditions. Thermophilic families, including *Thermoproteaceae*, show moderate USP investment, adapting to different stressors. The heterogeneity of structural motifs in *Halobacteriota*,* Methanobacteriota* and *Thermoproteota* suggests a correlation between domain complexity and stress diversity. High USP diversity enables robust responses to environmental variation, highlighting archaeal resilience and potential biotechnological applications in stress tolerance, industrial processes and biomedicine.

## Electronic supplementary material

Below is the link to the electronic supplementary material.

Supplementary Material 1

## Data Availability

No datasets were generated or analysed during the current study.
