# Supplementary material for: Exploring the structural landscape of universal stress proteins in Archaea
Source: World J Microbiol Biotechnol. 2025 Jun 11;41(6):196. doi: 10.1007/s11274-025-04439-y (PMC12159084; doi:10.1007/s11274-025-04439-y)
Supplement: Supplementary file 1 — Supplementary Material 1 [file 11274_2025_4439_MOESM1_ESM.docx]

Exploring the Structural Landscape of Universal Stress Proteins in Archaea

Matarredona, L., García-Bonete, M.J., Zafrilla, B., Esclapez, J.

Department of Biochemistry and Molecular Biology and Soil Science and Agricultural Chemistry, Faculty of Science, University of Alicante, Alicante, Spain.

Corresponding author: julia.esclapez@ua.es


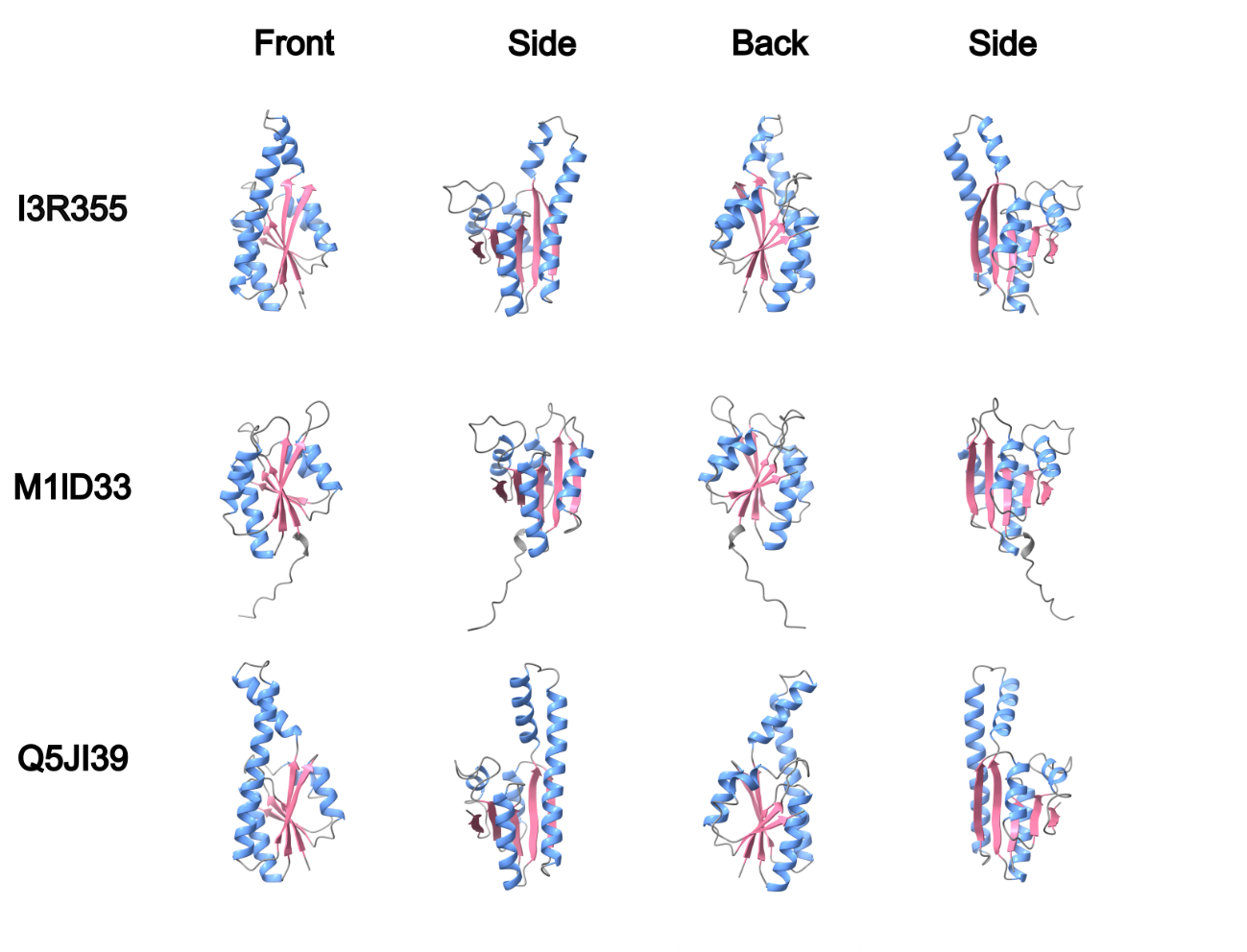


**Figure S1**. Secondary representation of single USP proteins for each group. Extra helix is observed in I3R355 and Q5JI39 but absence in M1ID33 which is more compact.


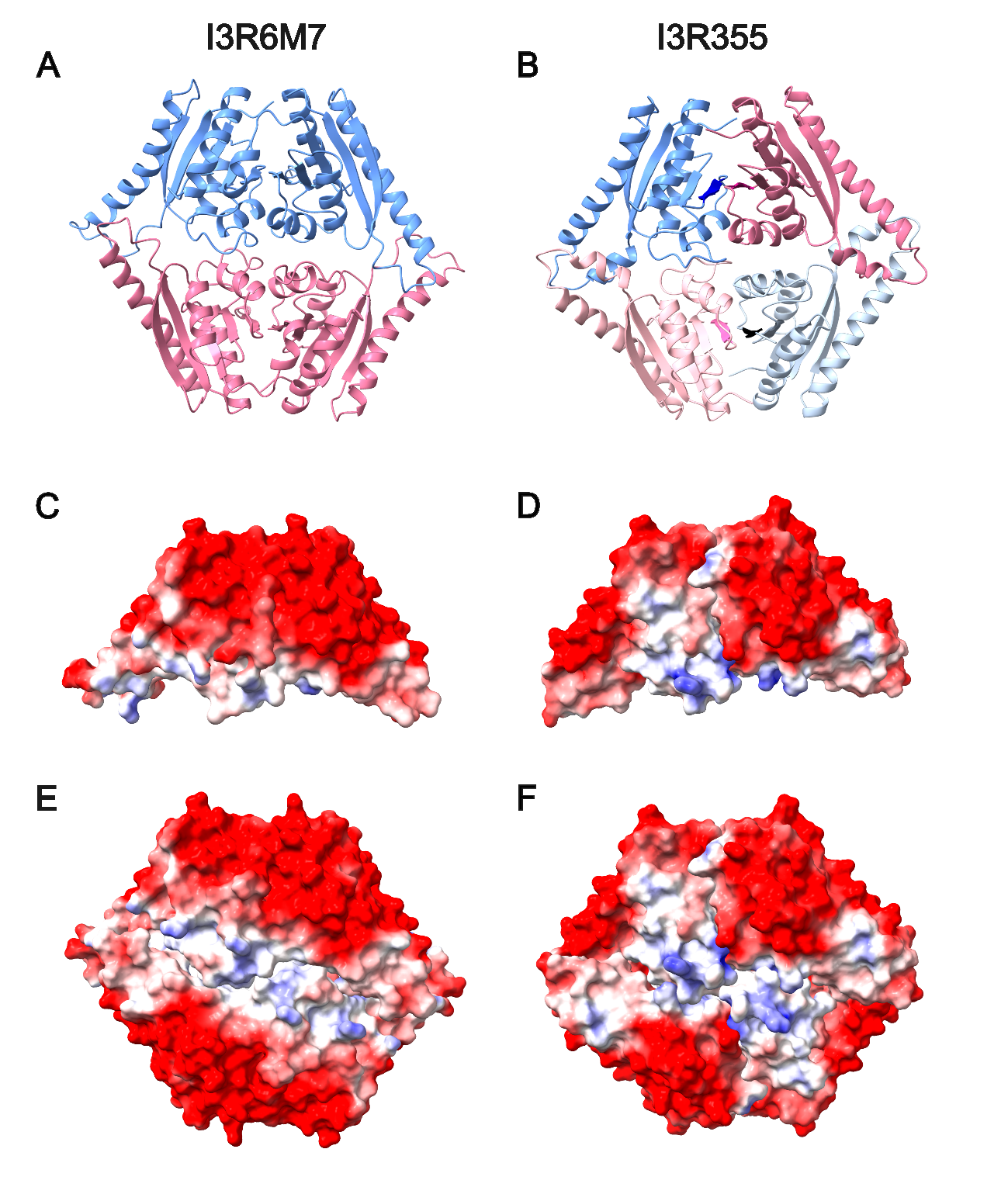


**Figure S2**. Oligomerization models of USP-UPS (I3R6M7) and single USP (I3R355) proteins. **A.** Dimerization of USP-UPS protein model (I3R6M7) generated by Alphafold. One structure is represented in blue, the other in pink. **B**. Tetramerization of single-UPS protein model, I3R355, generated by Alphafold, the four molecules are represented in pink, light pink, blue and light blue. **C** and **D**. Surface electrostatic potential of USP-UPS monomer and single-USP dimer. **E** and **F.** Surface electrostatic potential of USP-USP dimer and single USP tetramer. Red represents the negatively charge residues, blue the positively charge residues and the white the non-charge residues.
